# Outcomes of Therapeutic and Tectonic Penetrating Keratoplasty in Eyes with Perforated Infectious Corneal Ulcer

**DOI:** 10.4274/tjo.galenos.2018.06937

**Published:** 2019-04-30

**Authors:** Cezmi Doğan, Osman Şevki Arslan

**Affiliations:** 1 İstanbul University Cerrahpaşa Faculty of Medicine, Department of Ophthalmology, İstanbul, Turkey

**Keywords:** Cornea, abscess, therapeutics, penetrating keratoplasty

## Abstract

**Objectives:**

To evaluate the outcomes of penetrating keratoplasty performed for therapeutic and tectonic purposes in eyes with perforated infectious corneal ulcer.

**Materials and Methods:**

This retrospective study included 43 eyes of 43 patients who developed perforated infectious corneal ulcer of various etiological causes between June 2008 and January 2018. The patients were evaluated based on age and sex, follow-up time, presence of corneal perforation, pre- and postoperative visual acuity, postoperative graft transparency, complications, and infection recurrence.

**Results:**

The mean age of the 43 patients was 52.9±13.8 years. The mean follow-up time was 2.7±1.3 years. Preoperatively, the visual acuity of the eyes was at the level of hand motions or counting fingers; postoperative best corrected visual acuity ranged from hand motions to 0.7. Postoperative complications included hyphema in 8 patients (18.6%), elevated intraocular pressure in 14 (32.5%), posterior synechiae in 18 (41.8%), and cataract in 22 patients (51%). Therapeutic and tectonic success was achieved in 42 patients (97.6%). Postoperative graft transparency was observed in 35 patients (83.3%) within the 1-year follow-up period and in 27 patients (71.0%) at 2 years. Among 27 patients with graft transparency, 23 had bacterial and 4 had viral etiologies (p=0.52); 16 patients had perforations smaller than 1 mm and 11 had perforations 1-3 mm in size (p=0.2).

**Conclusion:**

Therapeutic-tectonic keratoplasty for perforated infectious corneal ulcer successfully restored globe integrity in 97.6% of cases. The rate of graft transparency was 71.0% at 2 years, with no effect of etiological agent or perforation size.

## Introduction

Corneal infections occasionally show resistance to medical treatment and may progress acutely to corneal perforation. Depending on the clinical course, interventions such as amniotic membrane transplantation, conjunctival flap shifting, cyanoacrylate tissue adhesive use, and therapeutic lamellar or penetrating keratoplasty may be performed.^1,2,3,4^ In patients with corneal abscess, the aim of therapeutic-tectonic penetrating keratoplasty (TTPK) is to remove the infected tissue, thus reducing the infection load, and to restore globe integrity in patients who develop corneal perforation. TTPK performed in patients with corneal perforation due to ulcerative infection has a high complication rate, which also indirectly increases the probability of corneal graft failure. The aim of this retrospective study was to evaluate the outcomes of TTPK procedures performed in eyes with perforated infectious corneal ulcer in our hospital.

## Materials and Methods

This study was conducted in the Cerrahpaşa Faculty of Medicine, Department of Ophthalmology in compliance with the Declaration of Helsinki. The study protocol was approved by the local ethics committee (07.01.2016).

We retrospectively evaluated the outcomes of TTPK procedures performed in our clinic between June 2008 and January 2018 in 43 eyes of 43 patients who developed perforated infectious corneal ulcer of varying etiology which was considered to threaten globe integrity and was resistant to medical therapy. Patients with non-infectious corneal ulcer were not included in this study. The patients’ age and sex, follow-up time, presence of corneal perforation, pre- and postoperative visual acuity, postoperative graft transparency, complications, and recurrence of infection were evaluated. Visual acuity was measured preoperatively and postoperatively using a Snellen chart and was expressed in decimal. Swabs were taken from the site of infection for microbiological diagnosis. The samples were routinely Gram stained and Giemsa stained, and were also cultured in blood agar, chocolate agar, and Sabouraud dextrose agar. Corneal material was sent for polymerase chain reaction analysis for herpes simplex in necessary cases. Patients with suspected bacterial etiology were started on topical vancomycin (50 mg/mL) and amikacin or ceftazidime (50 mg/mL) fortified drops (one drop per hour) as empirical therapy until a microbiological diagnosis was made. In addition, patients suspected of fungal keratitis were treated with topical fluconazole (2 mg/mL) or voriconazole (10 mg/mL) (one drop per hour) and oral fluconazole 200 mg/mL or voriconazole 200 mg/mL twice daily. This initial medical treatment was modified according to the microbiological results. In selected cases, special growth media were used for the diagnosis of mycobacteria and acanthamoeba. Chlorhexidine 0.02% was used in combination with 0.1% propamidine isethionate Brolene^®^ (Sanofi, UK) for the treatment of *Acanthamoeba* every hour until surgical intervention. After keratoplasty the medication was stopped.

Confocal microscopy was used for differential diagnosis when possible. Antimicrobial susceptibility testing of the isolated microorganisms was done with the disc diffusion method. When needed, B-scan ultrasonography was performed and repeated at regular intervals.

### Surgical Method

Eyes with corneal perforations less than 1 mm in size were first treated by applying a tissue adhesive. Patients for whom this method provided long-term globe integrity were excluded from the study. Patients whose clinical condition progressed despite treatment were prepared for emergent TTPK surgery. All surgeries were performed by the same surgeon (O.Ş.A.) under general anesthesia. The center of the cornea was marked with a marking pen when applicable. Trephination was performed so as to include all infected tissue as well as 0.5 mm of healthy tissue. The lesion did not affect the limbal region in any of the cases.

Recipient corneas were excised using a trephine. The trephination step was performed by making a preincision without applying excessive pressure and manually dissecting with scissors guided by this preincision. The anterior chamber was filled with viscoelastic material, and peripheral and posterior synechiae were released. Membranes formed in the pupillary area were peeled off, and hypopyon and fibrotic material in the anterior chamber were removed by washing. Peripheral iridectomy was performed in all cases. The anterior chamber and cornea were washed with 1% vancomycin in cases of bacterial keratitis, and were washed continuously with 0.2% fluconazole or 1% voriconazole until the donor graft was placed in cases of fungal infection.

The covered cornea technique was used in cases with extremely shallow anterior chamber with protruding anterior segment structures from the incision site as described in our previous study.^5^ Briefly, in the covered cornea technique, following corneal incision the recipient button was sutured to the recipient rim. Viscoelastic material was used to fill the anterior chamber and protect the endothelial side of the donor button, which was then sutured to the recipient rim. In the next step, the sutures between the recipient rim and recipient button were cut and the recipient button was removed through the unsutured segment of the donor button.

A graft 0.5 mm larger than the recipient bed was cut from the endothelial part of the donor cornea using a punch. The donor cornea was sutured to the recipient bed with frequent interrupted sutures or a combination of interrupted and running sutures. The corneal button was divided into two and sent to the relevant units for histopathological and microbiological assessment.

Fortified antibiotic therapy was administered postoperatively. Topical steroids were started at a dose varying between 3 and 6 drops daily, depending on the patient, and were gradually tapered. In fungal cases, topical cyclosporine was initiated in the first week, then topical steroid drops were added when no recurrence of keratitis was observed. Steroid therapy was started at low dose (twice daily) and gradually increased during the course of treatment. In cases of recurrent herpetic keratitis, systemic acyclovir 2 g/day was administered during the first postoperative week and continued at a dose of 800 mg/day for at least 1 year. In patients with inactive herpetic keratitis, acyclovir was started at a dose of 800 mg/day and continued at 400 mg twice a day for at least 1 year. Patients taking systemic acyclovir were regularly checked for kidney function and patients taking systemic antifungal medication were regularly checked for liver function. Topical antiviral therapy was not administered to patients who underwent TTPK for herpetic keratitis. For all patients, antiglaucomatous drops were added to the treatment when necessary. Loose sutures were removed immediately.

### Statistical Analysis

A Fisher’s exact test or Yates correction test was used to compare the ratios according to the number of the samples. P values below 0.05 were considered statistically significant. SPSS (version 20.0) was used for all statistical analyses.

## Results

**Figure 1:**
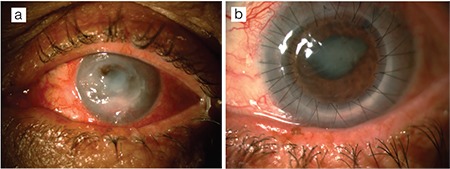
A case of bacterial keratitis. a) A central corneal perforation of 2-3 mm is evident and the anterior chamber is indistinct. b) At postoperative 1 month, the cornea is clear, there is no sign of recurrent infection, posterior synechia is present, and the peripheral iridectomies in the superotemporal and nasal quadrants are patent

**Figure 2:**
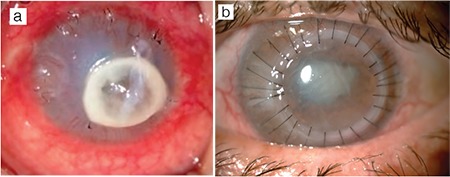
A case of fungal keratitis. a) Paracentral corneal perforation of 2-3 mm is evident. Emergent amniotic membrane transplantation was performed to preserve the integrity of the globe. During follow-up, the amniotic membrane dissolved and a portion can be seen covering the ulcer surface; sutures remaining from the membrane transplantation are visible in the peripheral cornea. b) At postoperative 1 month, corneal edema, posterior synechia, and cataract are apparent

The patients were evaluated in terms of age and sex, duration of follow-up, size of corneal perforation, pre- and postoperative visual acuity, postoperative graft transparency, anterior chamber status, complications, and recurrence of infection. Forty-three eyes of 43 patients (31 males, 12 females) were included. The mean age was 52.9±13.8 years. The mean follow-up time was 2.7±1.3 years (range, 7 months - 7 years). Mean graft dimensions varied between 6.5 mm and 8.00 mm depending on the size of the infectious corneal ulcer. Preoperatively, visual acuity was at the level of light perception, hand motion, or counting fingers; postoperatively, the best corrected visual acuity ranged from hand motion to 0.7 (Table 1). Corneal perforation (positive Seidel test) was present in all patients preoperatively. Size of the corneal perforation was less than 1 mm in size in 24 patients (group 1; 55.8%) and 1-3 mm in 19 patients (group 2; 44.2%) (Figures 1 and 2).

**Figure 3:**
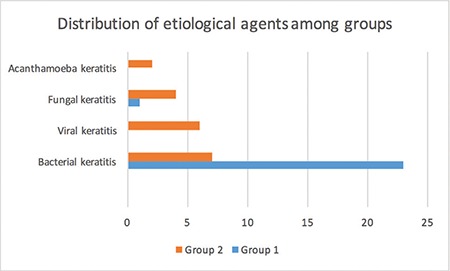
Distribution of etiological agents among groups. Size of the corneal perforation was less than 1 mm in group 1 and 1-3 mm in group 2

Infectious agents detected microbiologically included bacteria in 30 patients (69.7%), virus (positive herpes polymerase chain reaction test) in 6 patients (14%), fungi in 5 (*Candida in 1 case, Aspergillus* in 2 cases, and Fusarium in 2 cases) patients (11.6%), and *Acanthamoeba* in 2 patients (4.6%) (Figure 3). Direct bacteriological examination revealed gram-positive bacilli in 6 eyes, gram-positive cocci in 16 eyes, and gram-negative bacilli in 8 eyes. Among 30 eyes with bacterial keratitis, only 12 eyes (40%) had culture positivity, which consisted of coagulase-negative staphylococci in 3 eyes,* Staphylococcus aureus* in 2 eyes, *Streptococcus pneumoniae* in 2 eyes, Streptococcus viridans in 1 eye, *Pseudomonas aeruginosa* in 3 eyes, and Escherichia coli in 1 eye.

Anterior chamber reaction with 2+/3+ cells and flare was present postoperatively and fibrinoid membrane formation was observed in some patients. In patients suspected of infection recurrence, a sample was collected from the anterior chamber for microbiological evaluation.

### Postoperative Complications

**Figure 4:**
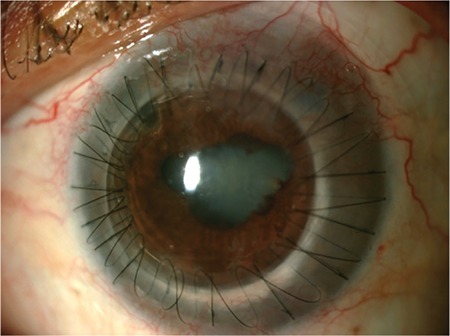
A patient with bacterial keratitis underwent therapeutic-tectonic penetrating keratoplasty due to a 2-3 mm corneal perforation. Loose sutures, posterior synechia, and cataract were observed at postoperative 2 months

Eight patients (18.6%) had varying degrees of hyphema in the anterior chamber postoperatively. The hyphema was cleaned by washing the anterior chamber in only 2 patients (4.6%); in the remaining patients, it regressed spontaneously with medical treatment. Fourteen patients (32.5%) had elevated intraocular pressure which was controlled with antiglaucomatous treatment. None of the patients had elevated intraocular pressure refractory to treatment or required glaucoma surgery. Permanent posterior synechia formed in 18 patients (41.8%). Twenty-two patients (51%) developed cataracts (Table 2) (Figure 4).

Therapeutic and tectonic success was achieved in 42 patients (97.6%). Recurrence was observed in only 1 patient in the postoperative period. This was a case of fungal keratitis, and the etiological agent was identified as *Candida*. Postoperative graft transparency was observed in 35 patients (35/42, 83.3%) during the 1-year follow-up period and in 27 patients at 2 years (27/38, 71.0%). Among 27 patients with graft transparency, 16 were in group 1 and 11 were in group 2. No significant difference was detected between the groups in terms of graft transparency at 2 years (p=0.2). However, development of cataract (p=0.003), synechiae posterior (p=0.0002), and glaucoma (p=0.02) after keratoplasty were significantly more common in the group 2. When graft transparency at 2 years was compared according to the etiological agent, no significant difference was detected between bacterial and viral keratitis (p=0.52) (Table 3).

## Discussion

Although penetrating keratoplasty is easier to learn and practice compared to lamellar techniques (deep anterior lamellar keratoplasty, Descemet membrane endothelial keratoplasty), performing penetrating keratoplasty in patients with perforated infectious corneal ulcer requires good surgical experience, surgical technique, and management. Intraoperative complications of penetrating keratoplasty are expected to occur at a higher rate in cases of perforated infectious corneal ulcer, possibly even leading to loss of the eye. Unlike conventional penetrating keratoplasty, postoperative success in cases of perforated infectious corneal ulcer is significantly influenced by the technique used by the surgeon (e.g., in choosing corneal graft diameter, performing peripheral iridectomy, and maintaining hemostasis) in addition to intraoperative complications. Because the intraocular structures may protrude through the perforation site or be damaged by the instrument when the recipient cornea is being excised using a trephine, the trephination step was performed by making a preincision without applying excessive pressure and manually dissecting with scissors guided by this preincision. In order to increase surgical success, effort was made in particular to open anterior synechiae, precautions were taken to enable fluid passage between the anterior and posterior chambers, and in necessary cases, a frequent symmetrical or asymmetrical (more frequent sutures in some quadrants) suture technique was used to prevent postoperative wound leakage. In eyes with a greater possibility of postoperative wound leakage, running sutures were applied in combination with interrupted sutures. Running sutures were placed between the interrupted sutures in order to achieve better donor-recipient apposition.

All of our cases had perforated corneal ulcers of varying size. Emergency amniotic membrane transplantation or tissue adhesive was used in some cases in addition to medical treatment in order to preserve integrity of the globe and reduce inflammation. Particularly in patients with corneal perforations smaller than 1 mm (group 1), a tissue adhesive was applied in addition to medical treatment, and only those who exhibited progression despite this approach were operated.

Cases of bacterial keratitis usually respond quickly to medical treatment and require TTPK less often. Ti et al.^8^ determined that *Pseudomonas aeruginosa* is the bacterial agent more commonly responsible for corneal perforation, though gonococcal and atypical mycobacterial infections may also require therapeutic keratoplasty.^6,7^ In the present study, the majority of patients who underwent TTPK due to perforated infectious corneal ulcer had bacterial keratitis, representing 69.7% of our study group. *Pseudomonas* was detected in the cultures of 3 patients, and these accounted for 3 of the 19 cases with large corneal perforations (group 2). The fact that these patients were initially treated at other centers and referred to our clinic at a late stage resulted in higher rates of TTPK for perforated infectious corneal ulcers due to bacterial agents. Nearly all of the patients who were operated had started treatment at a different center and were later referred to us. No recurrent infections were observed in the bacterial keratitis cases in the postoperative period.

We had 5 cases of perforated infectious corneal ulcer due to fungal keratitis, accounting for 11.6% of our TTPK procedures. Of these, the etiological diagnosis was *Candida* in 1 case, *Aspergillus* in 2 cases, and *Fusarium* in 2 cases. Difficulty finding antifungal drugs, the low drug susceptibility of some fungus types, and delayed etiological diagnosis lead to progression despite antifungal therapy, and necessitate emergent tectonic keratoplasty to preserve globe integrity. Since fungal keratitis has poor prognosis, therapeutic lamellar keratoplasty is considered urgent in order to prevent deeper corneal layers from being affected while the infection is still superficial. However, in our cases, Descemet’s membrane was affected and there was corneal perforation. Infection recurred and progressed to endophthalmitis in one of these 5 patients postoperatively; intravitreal antifungal therapy was able to preserve integrity of the globe. This was the only patient in our study with recurrent infection. There were few fungal cases in our study; Xie et al.^9^ reported a 15.4% rate of infection recurrence after keratoplasty for fungal keratitis. Key steps in managing recurrent infection are washing the anterior chamber with antifungal drugs intraoperatively and avoiding the use of steroids in the early postoperative period. A patient’s systemic condition, particularly uncontrolled high blood sugar, also increases the recurrence of fungal infection. Said et al.^10^ and Sedghipour et al.^11^ found that penetrating keratoplasty was effective in the treatment of fungal keratitis, but noted a high rate of postoperative immune rejection (27.2%-29.6%).^8^

Herpetic keratitis recurrence has been reported at 20% in the literature.^12^ Our cases did not have any recurrence during the follow-up period.

Two patients in our study underwent TTKP due to corneal perforation secondary to *Acanthamoeba* keratitis. No recurrence was observed during postoperative follow-up. However, the donor cornea showed decompensation and loss of transparency within 1 year of keratoplasty. In addition, their eyes also developed cataract and glaucoma which was controlled with medication. Despite the limited number of patients with *Acanthamoeba* keratitis in our study, a previous study determined recurrent infection to be among the most common complications after TTKP (40%).^13^ Of the patients who did not have recurrent infection in that study, 36% required multiple keratoplasty and 32% developed glaucoma.^13^

Postoperative graft transparency declines as the average graft size exceeds 8 mm and the graft approaches the limbus. The most important factor affecting graft diameter is the size and location of the infectious corneal ulcer. In the present study, graft dimensions were determined based on the dimensions of the infectious corneal ulcer and ranged from 6.5 mm to 8.00 mm. Graft transparency varies between 23-84.6% in the literature and we had similar outcomes, with 83.3% at 1 year and 71% at 2 years.^8,9,14,15,16,17^

When the patients were classified according to corneal perforation size, we observed that the complication rate increased with perforation size (Table 2). However, there was no significant difference between the groups in terms of graft transparency at 2 years despite higher complication rates (cataract, synechiae posterior, and glaucoma) in group 2.

Perforations associated with fungal and viral keratitis etiologies were predominant in the large perforation group (group 2), while smaller perforations (group 1) comprised mostly bacterial keratitis cases. Although we had few cases with viral etiology, no significant difference was detected in terms of graft transparency at 2 years between the bacterial and viral groups.

### Study Limitations

The limited number of patients and retrospective design of the study are two of its limitations. Since most of the patients were referred to our department after starting treatment in a different center, bacterial culture did not yield positive results to show the causative microorganism in most of the patients who had corneal perforation due to keratitis. Thus, their treatment was dependent on the results of direct microscopic examination.

## Conclusion

TTPK in patients with perforated infectious corneal ulcer requires more surgical experience (with donor cornea diameter, peripheral iridectomy, hemostasis control) compared to conventional routine penetrating keratoplasty. Using TTPK to treat perforated infectious corneal ulcer, we achieved a 97.6% success rate in preserving the integrity of the globe and eliminating the infectious agent, and graft transparency was 71.0% at postoperative 2-year follow-up. Etiological agent did not seem to affect graft transparency at 2 years. In addition, although larger corneal perforations may have contributed to the development of more complications, perforation size was not associated with rate of graft transparency at 2 years.

## Figures and Tables

**Table 1:**
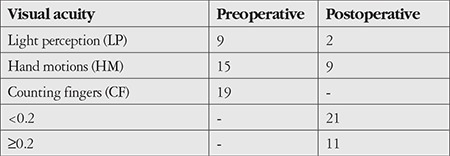
Preoperative and postoperative visual acuity levels

**Table 2:**
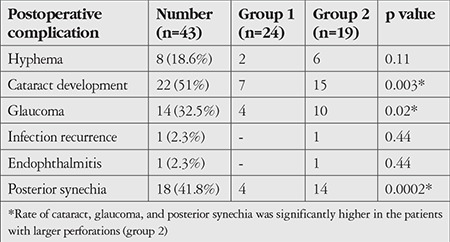
Postoperative complications according to size of corneal perforation

**Table 3:**
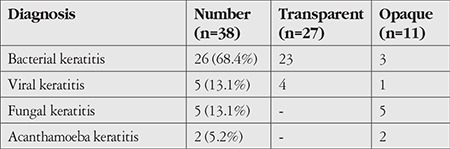
Graft transparency at 2 years according to the etiological agent
